# Proline codon pair selection determines ribosome pausing strength and translation efficiency in bacteria

**DOI:** 10.1038/s42003-021-02115-z

**Published:** 2021-05-17

**Authors:** Ralph Krafczyk, Fei Qi, Alina Sieber, Judith Mehler, Kirsten Jung, Dmitrij Frishman, Jürgen Lassak

**Affiliations:** 1 Department of Biology I, Microbiology, Ludwig-Maximilians-Universität München, München, Germany; 2 Institute of Genomics, School of Biomedical Sciences, Huaqiao University, Xiamen, China; 3 Department of Bioinformatics, Wissenschaftzentrum Weihenstephan, Technische Universität München, Freising, Germany

**Keywords:** Ribosome, Evolutionary genetics, tRNAs, Bacterial genetics

## Abstract

The speed of mRNA translation depends in part on the amino acid to be incorporated into the nascent chain. Peptide bond formation is especially slow with proline and two adjacent prolines can even cause ribosome stalling. While previous studies focused on how the amino acid context of a Pro-Pro motif determines the stalling strength, we extend this question to the mRNA level. Bioinformatics analysis of the *Escherichia coli* genome revealed significantly differing codon usage between single and consecutive prolines. We therefore developed a luminescence reporter to detect ribosome pausing in living cells, enabling us to dissect the roles of codon choice and tRNA selection as well as to explain the genome scale observations. Specifically, we found a strong selective pressure against CCC/U-C, a sequon causing ribosomal frameshifting even under wild-type conditions. On the other hand, translation efficiency as positive evolutionary driving force led to an overrepresentation of CCG. This codon is not only translated the fastest, but the corresponding prolyl-tRNA reaches almost saturating levels. By contrast, CCA, for which the cognate prolyl-tRNA amounts are limiting, is used to regulate pausing strength. Thus, codon selection both in discrete positions but especially in proline codon pairs can tune protein copy numbers.

## Abstract (web summary)

Ralph Krafczyk et al. demonstrate that both proline codon usage and prolyl-tRNA abundance affect mRNA translation rates of polyproline-motifs in bacteria. These results provide additional insight into sequence-specific translation regulation.

## Introduction

Proline has a set of characteristics that is not found in other proteinogenic amino acids. It is the only n-alkyl amino acid and thus has unique chemical properties. Its pyrrolidine ring makes proline conformationally rigid and thus it can shape protein structure: depending on its configuration—*cis* or *trans*—the binding axis rotation of amide bonds changes with major consequences for folding^1^. Peptide stretches enriched in prolines can even form a distinct type of secondary structure, the so called polyproline helix^2^. However, all these unique features come at a price. Not only is peptide bond formation with proline the slowest compared to all other proteinogenic amino acid^3–5^, but ribosomes can even be arrested when translating stretches of proline residues^6–8^. However, consecutive prolines occur frequently in eukaryotic and prokaryotic proteomes^9,10^. For example, in *Escherichia coli* every third protein contains at least one polyproline motif (PP-motif, at least diproline)^11^ and in *Streptomyces* species there is more than one PP-motif per protein on average^12^. The explanation for this apparent oddity is the existence of a ubiquitous elongation factor (termed EF-P in bacteria and a/eIF5A in archaea/eukaryotes) that alleviates ribosome stalling^13–16^. Nevertheless, EF-P cannot fully compensate for the translational burden caused by PP-motifs^11^. Intriguingly, bacteria can even benefit from ribosomal pausing by using it to regulate translation rates^14^. PP-motifs are enriched in inter-domain linker regions, which might promote correct folding, upstream of transmembrane regions, where they could facilitate correct insertion, and close to the protein N-terminus^11^. Here, similar to rare codons^17^, PP-motifs might be instrumental in generating a translational ramp and helping to avoid ribosome collisions^18^.

**Fig. 1:**
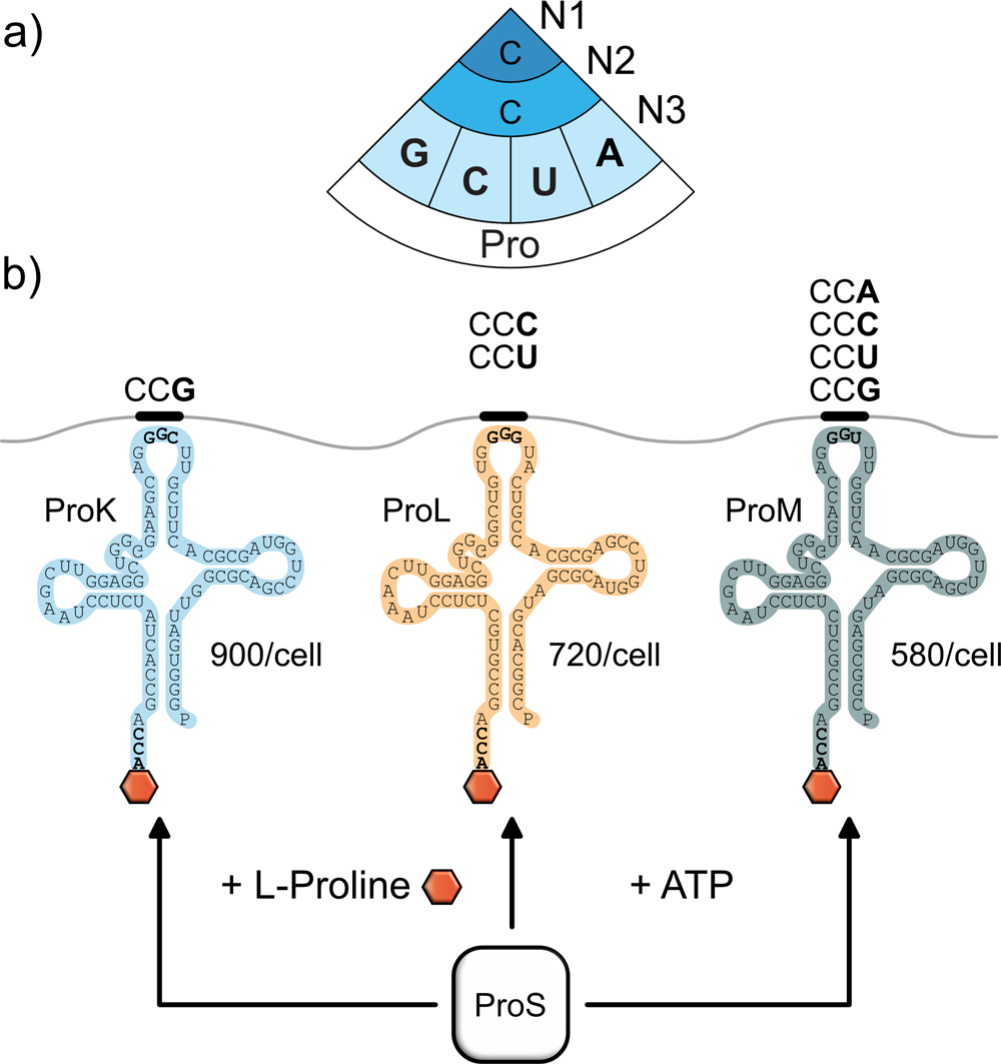
Diversity of proline codons and their corresponding tRNAs. **a** The genetic code contains four codons for proline: CCG, CCC, CCU, and CCA. **b** The three tRNAs ProK, ProL, and ProM recognize distinct sets of proline codons and exhibit different levels of abundance within the cell^34^. All three prolyl-tRNAs are charged by the prolyl-tRNA synthetase ProS.

It is well accepted that the amino acids bracketing PP-motifs influence the pausing strength^19–22^, thus representing a specific regulatory mechanism of translation. The role of proline codon choice, however, has not yet been investigated, although the incorporation speed of proline into the nascent chain differs significantly depending on which of the four codons (CCA/C/G/U) (Fig. 1a) and three tRNAs (ProK/ProL/ProM) are used (Fig. 1b)^5^. Here, we have comprehensively investigated how the interplay of codon choice and tRNA abundance affect the translation of PP-motifs.

## Results

### Distribution of proline codon pairs suggests their regulatory role in translation

**Fig. 2:**
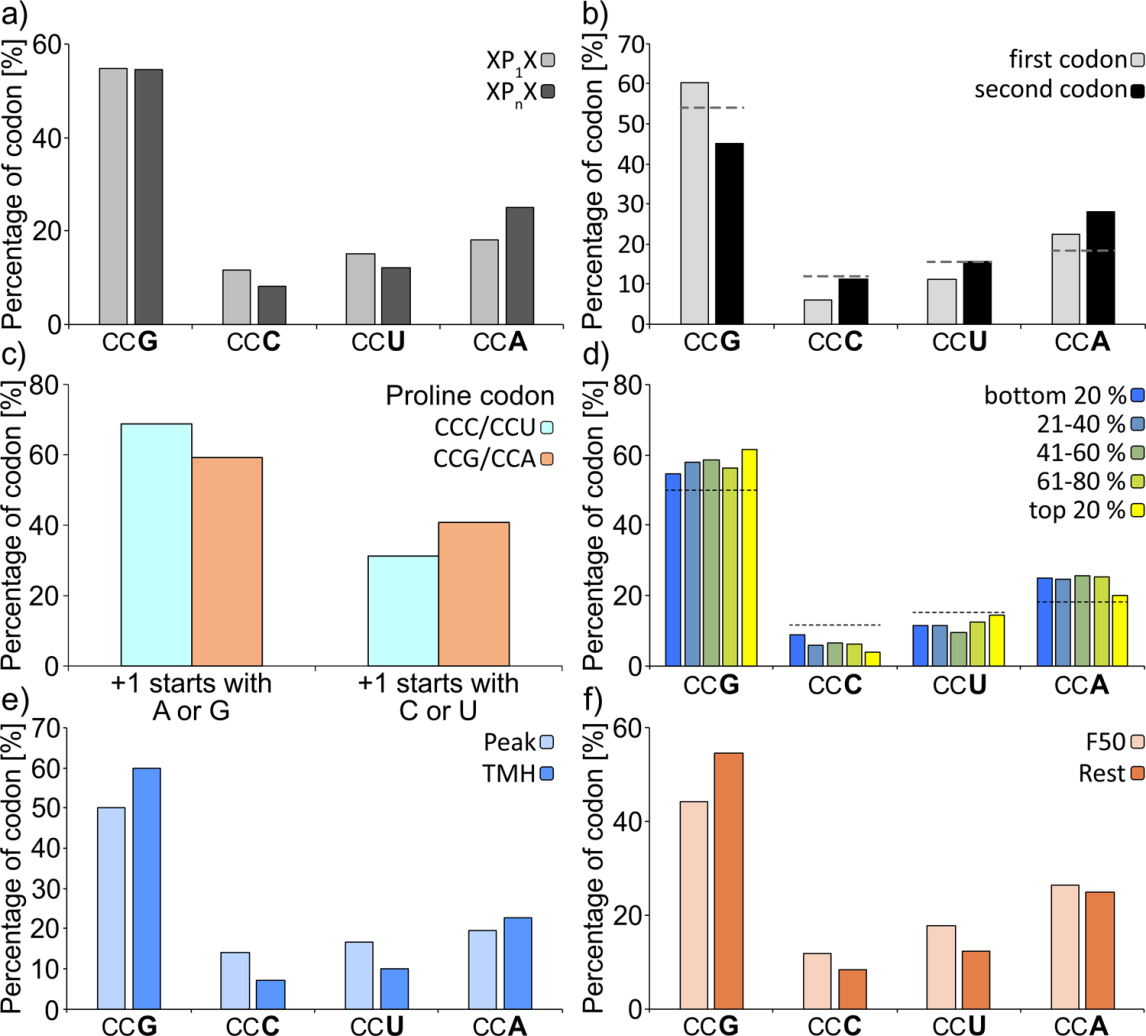
Bioinformatic analysis of proline codon bias in *E. coli*. **a** Codon usage of either single (XP_1_X) or consecutive (XP_*n*_X) prolines (with X being any amino acid except proline and *n* > 1). *p* value = 1.7e−30, chi-squared test. **b** Codon usage of the first and second proline in PP-motifs. Only PP-motifs with two consecutive proline residues were included in this analysis. The dashed lines indicate the codon usage for single prolines. *p* value < 2.2e−16, chi-squared test. **c** Codon usage for amino acids in the +1-position downstream CCC/CCU (cyan) or CCG/CCA (orange) encoded single prolines. *p* value < 2.2e−16, chi-squared test. **d** Correlation between proline codon usage in PP-motifs and translation efficiency from least efficiently translated proteins (dark blue) to most efficiently translated proteins (yellow). The dashed lines indicate the codon usage for single prolines. **e** Difference between proline codon usage of PP-motifs in the peak region (light blue, amino acids 49–59 from the TMH start where PP-motifs are enriched to facilitate the efficient insertion of TMH into the membrane) and TMHs (blue; transmembrane helices in which PP-motifs are depleted for proper folding of transmembrane segments^11^. *p* value = 0.13, chi-squared test. **f** Proline codon usage in PP-motifs in the first 50 codons (light orange) compared with the rest of proteins (orange). *p* value = 2.14e−7, chi-squared test.

**Fig. 3:**
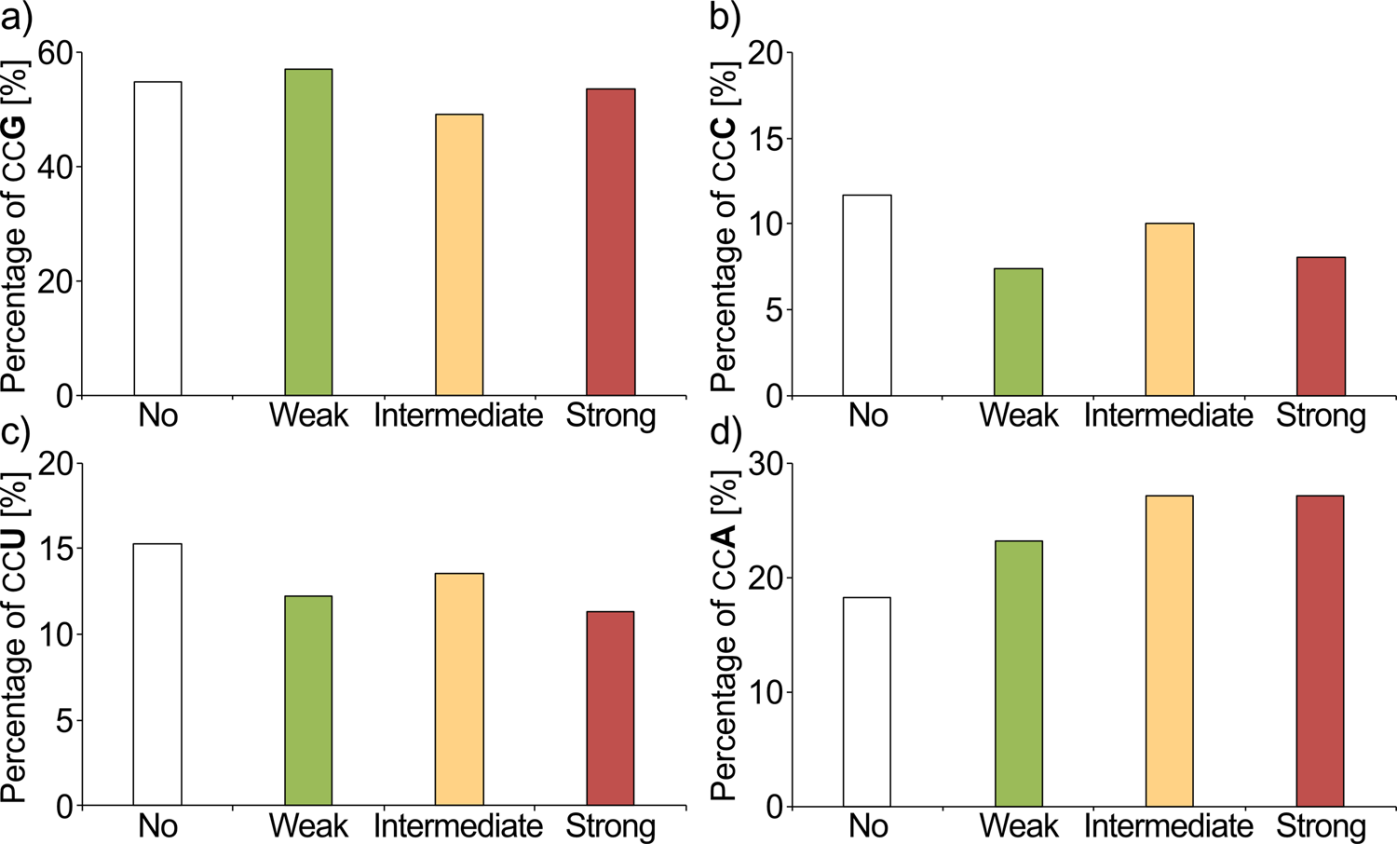
Codon usage in PP-motifs of different pausing strength. Pausing strength of PP-motifs depends on the upstream amino acid context^11,20,22^ resulting in weak, intermediate, and strong pausers. The pausing strength resulting from amino acid context is indicated by colored bars (no pausing—white; weak pausing—green; intermediate pausing—yellow; strong pausing—red). Codon usage in differently strong pausing motifs is shown for CCG (**a**), CCC (**b**), CCU (**c**), and CCA (**d**) codons. The difference is significant according to chi-squared test, *p* value = 4.2e−3.

Our study started with a bioinformatics analysis, in which we investigated whether codon usage differs between single prolines and proline pairs in the proteome of *E. coli* MG1655 (Figs. 2 and 3). We observed a depletion of CCC (8.1 vs. 11.6%) and CCU (12.3 vs. 15.3%) in codon pairs as compared to single prolines (Fig. 2a). Both of these codons delay diproline synthesis more (*t*_dip_[CCC]) = ~116.3 ms; *t*_dip_[CCU]) = ~71.4 ms) than the other two codons (*t*_dip_[CCA]) = ~66.7 ms; *t*_dip_[CCG]) = ~62.5 ms)^5^. Selection against slowly translating proline codon pairs is not restricted to *E. coli*: Out of 15 bacterial genomes with a broad range of GC-content values CCC and CCU are disfavored in 13 and 11 genomes, respectively (Fig. S1 and Supplementary data file S1). We next asked whether this bias might be related to codon order. Reportedly, an mRNA sequence of CC**C/U-C/U**CN promotes +1 ribosomal frameshifting, which is in principle counteracted by methylation of the corresponding isoacceptor tRNAs ProL and ProM at the 3′ side of the anticodon (m^1^G37)^23,24^. However, this modification cannot fully prevent ribosome slipping, as we could demonstrate with a bioreporter in vivo (Fig. S2). Accordingly, it would be plausible that the selective pressure on proline codon pairs is most pronounced for the first codon. Indeed, our analysis unveiled strong avoidance of both CCC and CCU at the first positions, while their occurrence at the second position matches their genome-wide usage (Fig. 2b). Further, the observed bias is not restricted to proline codon pairs but also to single prolines, as long as the downstream codon starts either with “C” or “U” (Fig. 2c).

Interestingly, the negative selection of CCC/U in proline codon pairs is not compensated by overrepresentation of CCG, being the most optimal codon in terms of diproline synthesis rates^5^. Instead, an enrichment of CCA (18.2 vs. 25%) was found. Ranking proteins with proline pairs according to their translation efficiency (Fig. 2d) revealed a preference for CCG in the top 20%. These findings imply a potential regulatory role of the relative CCA accumulation in PP-motifs, e.g., to slow down translation for proper membrane insertion or at the protein start to generate a translational ramp as a late stage of translation initiation thereby reducing ribosomal traffic jams^17^. In fact, non-CCG proline codons are enriched in these regions, further supporting the idea (Fig. 2e, f).

PP-motifs can be classified into “weak”, “intermediate”, and “strong” pausing motifs according to their interference with translation^11,20,22^. These differences result from the preceding amino acid. We were therefore interested whether specific proline codon biases exist within these subgroups of PP-motifs. Thus, we dissected PP-motifs accordingly (Fig. 3a–d and Supplementary data file S2). The most pronounced difference to single prolines was again the CCA usage (Fig. 3d). This codon represents 23.2% of all proline codons associated with weak pausing compared to 27.2% and 27.1% for intermediate and strong pausing, respectively. This difference is significant according to a two-sided *Z* test (*p* value = 7.0e−3). Thus, the differences between CCA and CCG in terms of pausing strength might be an additional mechanism to tune the translation efficiency.

### An in vivo reporter system to quantify translational pausing

**Fig. 4:**
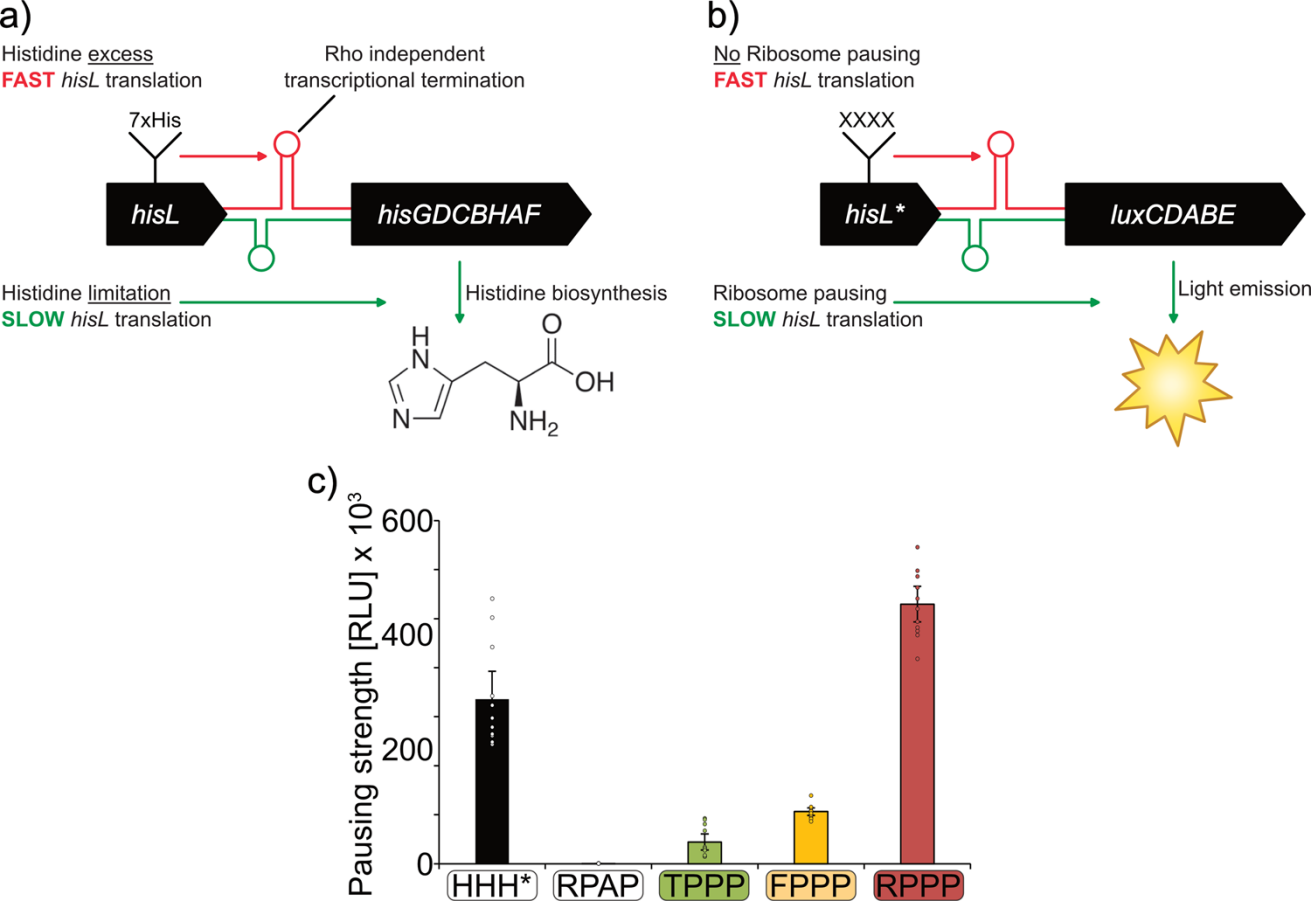
The His-pausing system for in vivo measurement of pausing strength. **a** Architecture of the histidine biosynthesis operon in *E. coli*. In its native state, the histidine biosynthesis gene cluster (*hisGDCBHAF*) is regulated by the His-leader peptide (*hisL*). This peptide contains seven consecutive histidines. At high histidine/histidyl-tRNA levels, translation efficiently proceeds through the His-leader peptide, resulting in the formation of an attenuator stem loop (red) that prevents transcription of the downstream genes. At low histidine and histidyl-tRNA levels translation is slowed down allowing for transcription and translation of the structural genes and synthesis of histidine (green). **b** Architecture of the His-pausing operon. An engineered His-leader peptide (hisL*) precedes the structural genes of the lux operon (*luxCDABE*). Here, His1 through His4 are exchanged by artificial sequence motifs (XXXX). In case of non-consecutive proline motifs (e.g., RPAP) there is no pausing, resulting in the formation of an attenuator stem loop (red) that prevents transcription of the downstream genes and low light emission. In the presence of motifs that contain consecutive prolines (e.g., RPPP) translation is slowed down allowing for transcription and translation of the structural genes and thus increased light emission (green). **c** Maximal luminescence emission at PP-motifs with increasing pausing strength. HisL*_Lux operons carrying a stop codon at the position corresponding to His4 (HHH*), non-consecutive (RPAP) or consecutive prolines of varying known pausing strength at the hisL* position (Weak: TPPP; green. Intermediate: FPPP; yellow. Strong: RPPP; red) were chromosomally integrated in *E. coli* BW25113 and tested for maximal luminescence emission. Threonine, phenylalanine, and arginine were encoded by ACC, TTT, and CGC, respectively. CCG was used as proline codon in all constructs. *n* = 12, Error bars indicate 95% confidence intervals.

In order to measure codon effects on translational efficiency, we established a reporter system that is capable of determining translational pausing strength within living cells. The system hijacks the attenuation mechanism of the histidine biosynthesis operon *hisGDCBHAF* (Fig. 4a)^25^. Here, translational speed of the preceding His-leader peptide (HisL) controls expression of the downstream structural genes^26^. Naturally this peptide contains seven consecutive histidines. When charged histidyl-tRNA is present in excess, ribosomes translate HisL non-stop, which in turn results in the formation of an mRNA attenuator stem loop that prevents transcription of *hisGDCBHAF*. When histidine concentrations are limiting, HisL translation is decelerated due to a lack of charged histidyl-tRNAs and an alternative mRNA stem loop is formed, which in turn permits transcription of the histidine biosynthesis genes. We fused the 5′ untranslated region (5′ UTR) of *hisGDCBHAF* as well as the preceding *hisL* with the *luxCDABE* operon of *Photorhabdus luminescens*^27^ and integrated the resulting construct via single homologous recombination into the *E. coli* chromosome (Fig. 4b)^28,29^. Monitoring of light emission over 16 h of growth showed a maximal output of only around 500 RLU, demonstrating that almost no pausing takes place under standard growth conditions in complex medium (LB). This was expected as LB contains about 1 mM of histidine^30^, which means an excess of about 100-fold^31^.

To assess the potential of our reporter to measure ribosome pausing we generated HisL variants encompassing PP-motifs of varying strength (Fig. 4c). Specifically, we substituted His1 through His4 by TPPP, FPPP, or RPPP being representatives of weak, intermediate, and strong pausers, respectively^22^. As a positive control, we placed a stop codon in the position corresponding to His4. As a negative control, we chose RPAP, which does not reduce translational speed^14^. As codon for alanine, we selected GCG being highly similar to the proline codon CCG. This choice was made to minimize putative effects of mRNA structural alterations.

To delineate codon effects from those caused by the peptide sequence all prolines were encoded only by CCG. The maximal light output of the corresponding *E. coli* strains ^RPAP^CC**G**, $^{{\underline{\rm{T}}}{\rm{PPP}}}{\rm{CC}}{\bf{G}}$, $^{{\underline{\rm{F}}}{\rm{PPP}}}{\rm{CC}}{\bf{G}}$, and $^{{\underline{\rm{R}}}{\rm{PPP}}}{\rm{CC}}{\bf{G}}$ increased from 390 RLU to 44,000 RLU to 106,000 and 530,000 RLU, respectively (Fig. 4c). The results obtained here perfectly match published datasets based on completely different experimental principles^14,19,20,22,32^. Accordingly, the outcome of our assay is a result of ribosome pausing that is determined by sequence identity but not mRNA structure. Notably, the positive control HHH* reached a maximum RLU of 336,000, which was in the same range as the reporter activity of the RPPP construct. We can therefore conclude that ribosome pausing induced by strong stallers is comparable to a stop caused by a termination signal.

Of particular interest is, that the measurements were conducted in an *E. coli* wild-type strain where stalling at consecutive prolines is alleviated by EF-P^14^. Thus, we have a tool in hand to determine pausing strength in vivo. Using the system, we unambiguously demonstrate that the burden associated with PP-motifs is an inherent translational feature and explains the strong selective pressure causing the proteome shaping^11^.

### Codon choice modulates pausing strength at consecutive proline motifs

**Fig. 5:**
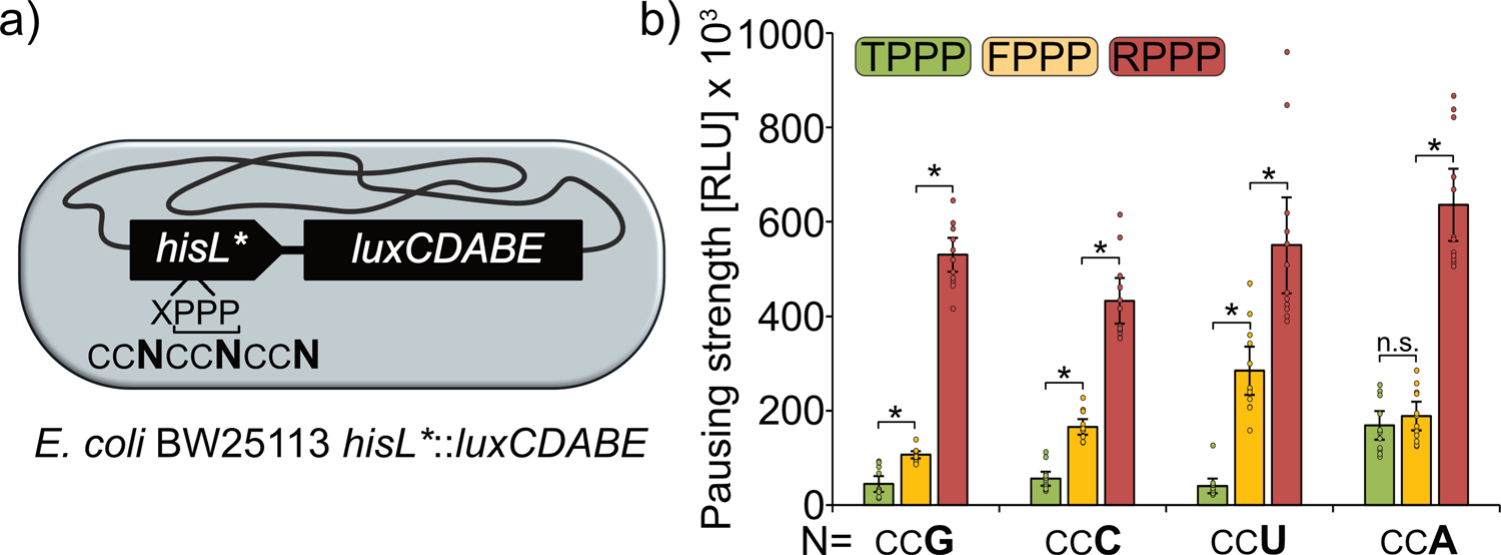
Codon-dependent pausing strength at weak, intermediate, and strong PP-motifs. **a** Genomic organization of the HisL*_Lux reporter. Synthetic His-Leader peptides (HisL*) preceding the *lux* genes (*luxCDABE*) were genomically integrated at the *his*-locus. In hisL*, His1 one was replaced by a variable amino acid (X) to modulate pausing strength^16^. His2 through His4 were replaced by proline. In this regard several reporter strains (Supplementary data file S3) were generated with hisL* varying in the proline codon usage and are denoted as $^{{\underline{\rm{X}}}{\rm{PPP}}}{\rm{CC}}{\bf{N}}$ where the underlined X designates the preceding amino acid and the bold **N** designates the wobble base used for encoding the proline residues. **b** HisL*_Lux carrying PP-motifs of varying pausing strength (weak—TPPP: green; intermediate—FPPP: yellow; strong—RPPP: red) with different proline codon usage were chromosomally integrated in *E. coli* BW25113 and tested for maximal luminescence emission. *n* = 12, Error bars indicate 95% confidence intervals. Data for CCG codons are duplicated from Fig. 5 for better overview. Statistically significant differences according to unpaired two-sided *t*-tests (*p* value < 0.05) are indicated by asterisks.

To investigate whether the statistical tendencies of codon usage in PP-motifs can be attributed to physiological differences we conducted a systematic in vivo analysis. To this end we constructed a series of 4 × 4 HisL*_Lux reporter strains (Fig. 5a and Supplementary data file S3). Utilizing the strong pauser RPPP, CCG, CCA, and CCU were indistinguishable from each other, each producing a maximal light output of over 525,000 RLUs (Fig. 5). Only with CCC codons we found around 1.2-fold reduced maximal light emission. When testing a motif with intermediate strength (FPPP) a different pattern was obtained. In this case, CCU stretches produced significantly more light than the other codons, whereas emission using CCG was significantly decreased. CCC and CCA ranged in the middle of both. Interestingly, the most pronounced effect of codon choice on pausing strength occurred with the weak pauser TPPP. The luminescence with $^{{\underline{\rm{T}}}{\rm{PPP}}}{\rm{CC}}{\bf{A}}$ was significantly elevated by at least threefold compared to the strains encoding $^{{\underline{\rm{T}}}{\rm{PPP}}}{\rm{CC}}{\bf{C}}$, $^{{\underline{\rm{T}}}{\rm{PPP}}}{\rm{CC}}{\bf{G}}$, or $^{{\underline{\rm{T}}}{\rm{PPP}}}{\rm{CC}}{\bf{U}}$. Notably, such an increase is equivalent to a step in the pausing strength from weak to intermediate pausing (Figs. 5b and S3A). This result is also in perfect agreement with our genome scale analysis (Fig. 3a–d) and explains the strong selection against CCA in weak pausers. On the other hand, the bias in favor of CCA in intermediate and strong pausers might be attributed to a regulatory role that requires a further slowdown of translation. To exclude that the observed effects derive from mRNA structure alterations, we conducted another analysis utilizing a second reporter series XPPP with X being N (AAC) for weak, L (CTG) for intermediate and W (TGG) for strong^22^ (Fig. S3B, C). Expectedly, the activities are congruent with the T/F/RPPP derived data including the CCA effect in the weak pausing context. Taken together, these results demonstrate that codon choice in PP-motifs is capable of influencing ribosome pausing.

### tRNA abundance influences pausing strength at all proline codons

The variations in proline codon bias of PP-motifs of varying strength, particularly the one of CCA, raised the question whether tRNA abundance might contribute to pausing strength. In *E. coli*, three tRNAs—ProK, ProL, and ProM—are responsible for decoding of proline codons (Fig. 1). ProM represents a general tRNA that is capable of recognizing them all^33^, while ProL and ProK are more specialized and decode CCC/U and CCG, respectively^5^. These differences have a quantitative effect on the reading probabilities of the individual codons. Taking the copy numbers of ProK (900/cell), ProL (720/cell), and ProM (580/cell) into account, CCG has the highest number of the corresponding tRNAs (900 + 580 = 1480/cell)^34^ and thus matches very well to the general codon usage in the *E. coli* genome, where more than 50% of all prolines are encoded by CCG (Fig. 2). CCA is the other extreme, being recognized solely by ProM and accordingly only 580 tRNA copies per cell are available for translation.

**Fig. 6:**
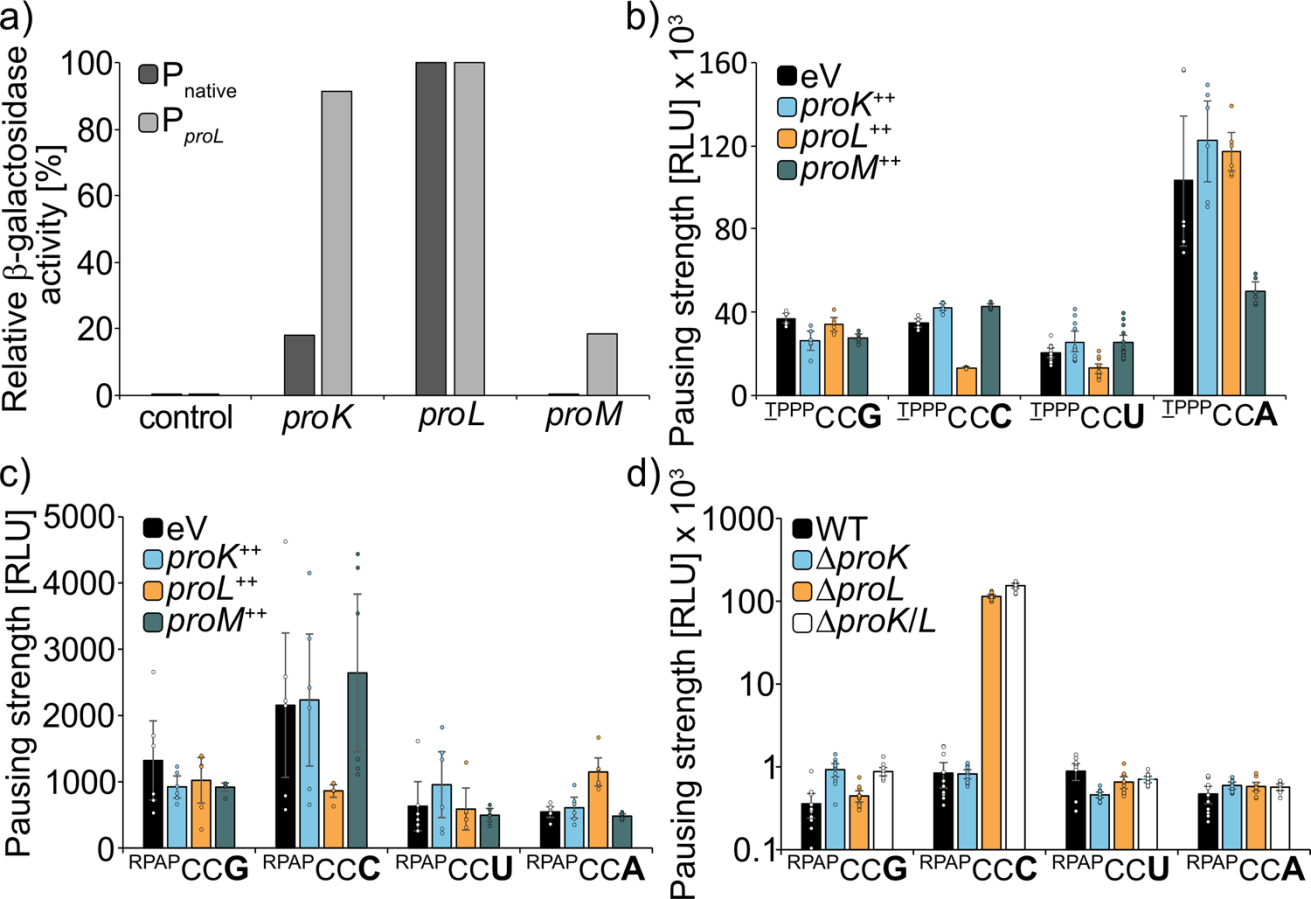
Influence of prolyl-tRNA copy number on the codon-dependent pausing strength at PP-motifs. **a** Approximation of *E. coli* BW25113 cells carrying the weak HisL*_Lux operon (TPPP) with different proline codon usage were transformed with pBBR1 MCS4-*lacZ* plasmids encoding ProK, ProL, or ProM under the control of their corresponding native promoters. *n* = 4. **b** *E. coli* BW25113 cells carrying the weak HisL*_Lux operon (TPPP) were transformed with pBBR1 MCS4-*lacZ* plasmids encoding for ProK, ProL, or ProM under control of P_*proL*_ and tested for bioluminescence emission. *n* = 6. **c** *E. coli* BW25113 cells carrying the “non-PP” HisL*_Lux operon (RPAP) were transformed with pBBR1-MCS4-*lacZ* plasmids encoding for ProK, ProL, or ProM under control of P_*proL*_ and tested for bioluminescence emission. *n* = 6. **d** The “non-PP” HisL*Lux operon (RPAP) was genomically integrated in *E. coli* BW25113 deletion strains lacking either *proK* (Δ*proK*), *proL* (Δ*proL*), or both (Δ*proK/L*) and cells were tested for bioluminescence emission. *n* = 12, Error bars indicate 95% confidence intervals.

To assess an effect of prolyl-tRNA copy numbers on pausing strength, we unbalanced the native ratios in favor of either ProK, ProL, and ProM (ProX^++^) by ectopically expressing them from P_*proL*_. Beforehand, the 5′ upstream sequences of *proK* (5′_*proK*_), *proL* (5′_*proL*_), and *proM* (5′_*proM*_) were tested on promoter activity, by generating an artificial operon with *lacZ* (Fig. 6a). As expected, no β-galactosidase activity could be measured when utilizing 5′_*proM*_, as *proM* is part of the *argX* polycistronic operon (*argX_hisR_leuT_proM*)^35,36^. From the remaining two regions—5′_*proK*_ and 5′_*proL*_—the latter gave a higher reporter signal and was therefore chosen as constitutive promoter for all three prolyl-tRNAs.

The effect of tRNA copy number increase was first assessed in the four reporter strains which harbor a HisL-TPPP variant each encoded by a series of one of the four distinct proline codons ($^{{\underline{\rm{T}}}{\rm{PPP}}}{\rm{CC}}{\bf{G}}$, $^{{\underline{\rm{T}}}{\rm{PPP}}}{\rm{CC}}{\bf{C}}$, $^{{\underline{\rm{T}}}{\rm{PPP}}}{\rm{CC}}{\bf{U}}$, $^{{\underline{\rm{T}}}{\rm{PPP}}}{\rm{CC}}{\bf{A}}$) (Fig. 6b). The CCG-specific ProK had a positive but only mild influence on pausing strength, solely when translating $^{{\underline{\rm{T}}}{\rm{PPP}}}{\rm{CC}}{\bf{G}}$. One plausible explanation is that the native copy number of 900/cell is already close to saturating levels and accordingly overexpression does not substantially add to pausing strength reduction. With ProL we observed significantly reduced pauses when testing $^{{\underline{\rm{T}}}{\rm{PPP}}}{\rm{CC}}{\bf{C}}$ and $^{{\underline{\rm{T}}}{\rm{PPP}}}{\rm{CC}}{\bf{U}}$, being again in line with the tRNA codon specificity. Interestingly, an increase in copy number of the general tRNA ProM had no major impact on reporter activity of the $^{{\underline{\rm{T}}}{\rm{PPP}}}{\rm{CC}}{\bf{G}}/{\bf{C}}/{\bf{U}}$ strains, indicative of a selection in favor of the more specialized tRNAs (ProK and ProL). Conversely, we saw a significant pausing strength reduction (>2-fold) for the $^{{\underline{\rm{T}}}{\rm{PPP}}}{\rm{CC}}{\bf{A}}$, which can only be decoded by ProM.

Second, to separate PP-motif specific effects from those also occurring only with single prolines, a reference reporter set encoding RPAP-HisL variants was included into our study (Fig. 6c). Here, the previously observed minor alleviating effect at CCG codons on translational pausing upon ProK overexpression was lost. On the contrary, an increase in the copy number of ProL still significantly reduced pausing strength at CCC codons, yet no reduction of reporter activity for ^RPAP^CC**U** was observed. CCC codons are reportedly translated the slowest^5^, which is in line with a general increase in luminescence compared to all HisL variants encoded by other proline codons. However, this does not explain the stimulatory effect on translational speed when overexpressing ProL: In their in vitro study on dipeptide synthesis with proline Pavlov et al. always employed bulk tRNA when measuring incorporation speed^5^. Accordingly, tRNA abundance effects were neglected. Our findings now indicate that the observed differences in dipeptide synthesis time might be partially due to ProL limitation. This idea is supported by the fact that the in vitro experiments in Pavlov et al. revealed CCU after CCC as the slowest codon to be decoded^5^.

The tRNA abundance effect that differs most between consecutive and single prolines is for CCA (Fig. 6b, c). While translational pausing is alleviated by a factor of around three upon overexpression of ProM, we hardly found any changes when analyzing the ^RPAP^CC**A** reporter. Thus, our findings provide a rationale for the CCA codon bias in PP-proteins.

Third, we performed the converse experiment by deleting the two non-essential tRNA genes *proK* and *proL*^33^, both individually—Δ*proK*, Δ*proL*—and in combination—Δ*proK/L*. These strains (Supplementary data file S3) were investigated on growth and cell morphology (Fig. S4) as well as on the effect they have on pausing strength (Fig. 6d). When analyzing the effects of *proK* and *proL* deletions on luminescence we saw the expected increase at ^RPAP^CC**G**, when *proK* is missing. The most striking results were obtained upon *proL* deletion. The light output at CCC significantly increased almost by a factor of 100, whereas translation of the ^RPAP^CC**U** reporter remained unaffected. This led us to conclude that the general tRNA ProM is a good decoder of CCU codons, as it can compensate for the lack of ProK. By contrast, the strong increase in pausing strength with CCC in Δ*proL* strains explains the necessity for a more specialized tRNA, which can outperform the ProM decoding capabilities at this specific codon. We therefore speculate that nature has evolved ProL predominantly to read CCC codons in order to compensate for its reduced translational speed. Additional reading of the “U” in the wobble position was acquired later, as a consequence of a mild advantage (Fig. 6b). This idea is also congruent with the identity of the ProL anticodon, which is GGG.

Taken together, we could show that tRNA abundance is a major driving force for the efficient translation of single and consecutive prolines.

### Proline codon choice finetunes protein copy number of the pH sensor CadC

Based on our results, we hypothesized that codon choice within PP-motifs can be used as a regulatory means to tune the pausing strength according to stoichiometric requirements. In this regard, counterselection of certain codons would occur in order to prevent modulation of the pausing strength predetermined by the amino acid context. To test this hypothesis, we investigated codon choice in the PP-motif of the transcriptional activator CadC.

**Fig. 7:**
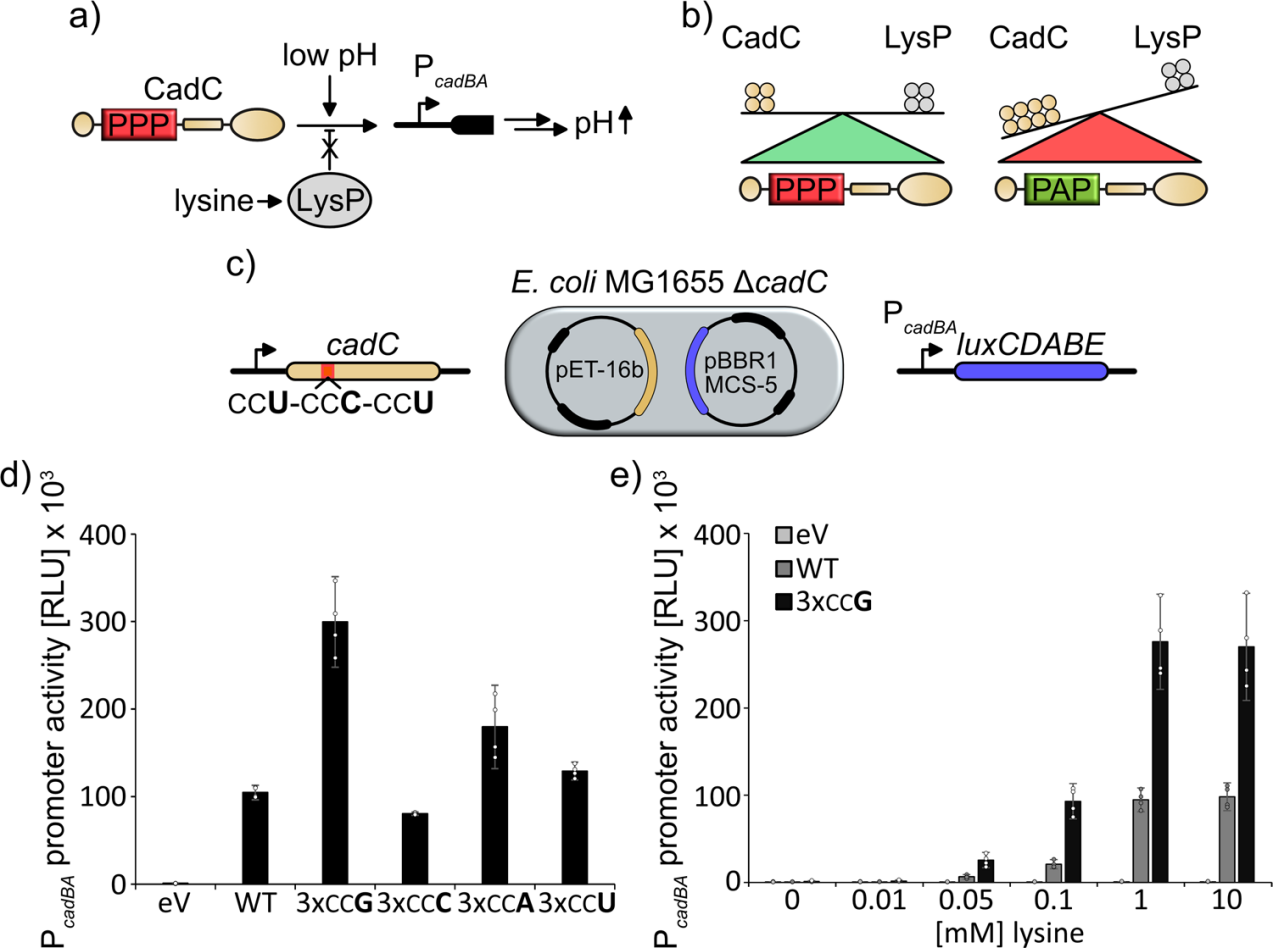
Codon choice modulates protein expression and ensures physiological protein stoichiometry of the Cad system. **a** The Cad system. CadC is a pH sensor that induces expression of its target genes at low pH by binding to the *cadBA* promoter (P_*cadBA*_). Expression of the corresponding gene products ultimately leads to an increase in pH. The lysine dependency of the acid stress response depends on stoichiometric expression of CadC and the co-sensor LysP. **b** The equilibrium of the protein copy numbers of CadC and LysP is ensured by a triproline motif within the CadC primary structure. Absence of the triproline results in deregulation of the acid stress response due to increased CadC copy number. **c** Reporter system used to test the *cadC* translation efficiency. *E. coli* MG1655 Δ*cadC* cells were transformed with pET-16B vectors encoding for wild type or proline codon-exchanged variants of CadC. Cells were cotransformed with pBBR1MCS-5 vectors carrying the *lux* genes under control of the P_*cadBA*_ promoter. P_*cadBA*_ promoter activity was assessed by measuring luminescence emission and used as a proxy for CadC copy number^14^. **d** P_*cadBA*_ promoter activity under inducing conditions (pH = 5.8; 10 mM lysine) upon expression of wild-type CadC or proline codon-exchanged CadC variants where all proline codons in the pausing motif have been substituted by the same codon. *n* = 4. **e** P_*cadBA*_ promoter activity at increasing external lysine concentrations. P_*cadBA*_ induction when *cadC* contains the natural codon composition is shown in dark gray. P_*cadBA*_ induction when *cadC* contains only CCG codons at the relevant PP-motif is shown in black. *n* = 4, Error bars indicate 95% confidence intervals.

CadC is a membrane-bound transcriptional regulator and part of the *E. coli* acid stress response^37–39^. The two external stimuli, mild acidic pH (<6.5) and lysine are needed to activate expression of the *cadBA* operon. While acidic conditions are sensed by CadC directly, lysine is recognized by a coregulator—the permease LysP. LysP directly interacts with CadC and a specific equilibrium between both proteins is crucial for an adequate transcriptional response (Fig. 7a)^14^. This equilibrium is strictly dependent on a triproline motif (aa120-122) within CadC^37^ that is decoded from CCU_P120_-CCC_P121_-CCU_P122_^14,32^ and preceded by a serine (TCG).

As expression of *cadC* from pET-16b leads to physiological protein levels and an adequate pH-stress response in *E. coli* MG1655 cells^14^, we generated plasmid-encoded CadC variants in which we unified the codons within the triproline motif (Fig. 7b). These were tested with a *lux* reporter controlled by P_*cadBA*_ (Fig. 7c)^40^. Upon monitoring the maximal light output during 16 h of growth in minimal medium under CadC inducing conditions (pH 5.8 and supplemented with 10 mM lysine) we detected a threefold increase in P_*cadBA*_ activity with CCG stretches in the CadC open reading frame while 3 × CCA, 3 × CCC, and 3 × CCU resulted in only subtle changes in light emission compared to the wild-type protein (Fig. 7d). As previously shown these changes in promoter activity reflect fluctuating copy numbers of the regulator^14^. Of note, only the changes between the CadC variants with 3 × CCA and 3 × CCG can be directly compared in terms of translation efficiency as a consequence of differences in tRNA abundance and codon anticodon pairing^5^. For the variants with 3 × CCC and 3 × CCU, the additional effect of ribosome slipping also causes decreased protein output.

To test for physiological repercussions of the elevated protein production with CadC^CCG^, we performed the same experiment again, but tested different lysine concentrations (Fig. 7e). In this setup both the wild type and the CadC^CCG^ variant reached the highest induction level at 1 mM lysine but the latter showed a threefold increased maximal light output. More importantly, CadC^CCG^ turned on *cadBA* transcription already at 100 µM lysine. This concentration, however, is insufficient for pH neutralization. Thus, codon choice within the CadC triproline motif is crucial to maintain an optimal ratio between CadC and LysP in order to achieve an adequate stress response.

## Discussion

The theory of codon bias postulates the correlation between preferred codons and abundances of their iso-accepting tRNAs^41^, thereby increasing translation efficiency^42^ and accuracy^43^. Although the “tRNA abundance” theory also applies to proline codons (Fig. S1)^34^, the strong correlation with incorporation velocities seems to be more important^5^. This explains, for example, why CCC is a rather neglected codon in *E. coli* as it interacts least efficiently with the tRNA^Pro^–EF-Tu–GTP ternary complex^5^. Moreover, a pair of CCC/U codons promotes ribosomal frameshifting (Fig. S2)^24^.

Generally, proline pairs are difficult to translate as they cause ribosome stalling^7^. Their frequent occurrence in nature points to a selective advantage that outweighs the concomitant translational burden^16^ and has even favored the emergence of a specialized elongation factor EF-P to aid in translation^14^. This advantage is due to the unique properties of polyprolines affecting protein structure^2^ and function^10^. Although there is an evolutionary trend to reduce the translational load, we have previously identified specific regions where pausing by PP-motifs is favored to limit translation rates and to facilitate proper membrane insertion and correct folding^11^.

Our previous work focused on the PP-motifs and their amino acid context. We have now extended our study to the transcript level, which led to several new insights into the relation between codon pairs and tRNA abundance.

First, we found that the codon bias in consecutive prolines differs significantly from that in single prolines, which helps to avoid slippery sequences (CCC/U-CCN) and to boost translation efficiency. Only in the regions where increased pausing time might be beneficial, such as the vicinity of the translational start and downstream of transmembrane helices, more slowly translating codons are favored (Fig. 2). Moreover, we have demonstrated the physiological importance of codon choice on one prominent example—the pH sensor CadC. Here, the silent mutation of prolines of CCU_P120_-CCC_P121_-CCU_P122_ into 3 × CCG led to a deregulation of the acid stress response as a result of an increased protein copy number. Thus, there is a concerted adjustment of both codon usage within PP-motifs and their amino acid context, in turn allowing for a precise adjustment of protein copy numbers. We note, that additional factors such as mRNA structure or stability might also contribute to this effect.

Second, we have uncovered the specific effects associated with isoacceptor tRNA^Pro^. Overall, we found that both overexpression and deletion of each individual prolyl-tRNA gene—*proK*, *proL*, or *proM*—affected translation at their cognate codons, regardless of their amino acid context. The most pronounced effect was observed with ProL and on CCC, whereas the benefit for the other target codon CCU was comparatively small. One reason for this might be the ProL anticodon—GGG, which could lead to different affinities between both of the recognized codons. Generally, dipeptide synthesis is slowest with CCC and CCU, which can also explain their scarceness in the genome. This rare usage was also one reason for having included *proL* into the pRARE plasmid in order to augment the yield and fidelity of heterologously produced proteins^44^. Our data now show that even under natural conditions ProL is limiting and thus increasing its copy number might have a positive effect on endogenously produced proteins (Fig. 6b, c). Especially, decoding of CCC benefits from ProL overproduction (Fig. 6) and thus heterologous expression of genes from GC-rich organisms such as *Streptomyces* species might lead to an increased yield. In this regard, it is notable that, e.g., *S. venezuelae* encodes a second copy of *proL*, presumably to circumvent this limitation (CCG: 52%, CCC: 43%, CCU: 3% CCA: 2%). Moreover, tRNA abundance explains also the selective pressure against CCA in weak PP-motifs. One might therefore speculate that recruitment of ProM to the ribosome is the rate limiting step in the weak context. Interestingly, increased copy number of ProM did not result in a decrease of ribosome pausing at any other codon than CCA. For CCC the reason might be in the poor interaction between the cmo5U34 modified base and the 3′ cytosine of the CCC codon. Besides that, even under control of P_*proL*_, proM was less efficiently transcribed than the other tRNAs, indicating that the relative titers compared to the more specialized ProK and ProL for translation of CCG/C/U were not as strongly affected. The preference for CCG for which the cognate tRNA levels are close to saturation is consistent with this idea (Fig. 6). Further, CCG is enriched in PP-motifs at the expense of CCA in the top 20% of proteins in terms of translation efficiency (Fig. 2). In general, CCG seems to be the “best” proline codon in bacteria, when it comes to translation efficiency of codon pairs. This also explains why especially this codon is avoided in the CadC proline codon triplet, as here an extremely low copy number is crucial for a regulated acid stress response^14^.

Thus, codon choice in proline codon pairs represents an elegant strategy to control translation efficiency and finetune protein copy numbers in bacteria.

## Material and methods

### Plasmid and strain construction

All strains, plasmids, and oligonucleotides used in this study are listed and described in Supplementary data files S3–S5, respectively. All kits and enzymes were used according to manufacturer’s instructions. Plasmid DNA was isolated using the Hi Yield® Plasmid Mini Kit from Süd Laborbedarf. DNA fragments were purified from agarose gels using the Hi Yield® Gel/PCR DNA fragment extraction kit from Süd Laborbedarf. All restriction enzymes, DNA modifying enzymes, and the Q5® high fidelity DNA polymerase for PCR amplification were purchased from New England BioLabs.

The pNPTS-138-R6KT_*hisL*_*luxCDABE* vector was generated by amplification of *hisGDCBHAF* operon leader peptide *hisL* from *E. coli* BW25113 genomic DNA and ligation into pNPTS-138-R6KT_P_*BAD*__*luxCDABE* after restriction with *Sph*I and *Nco*I. All variants of *hisL* (hisL*, Supplementary data file S4) were generated by overlap extension PCR with mutagenized primers (Supplementary data file S5) from pNPTS-138-R6KT_*hisL*_*luxCDABE* and subsequent cut/ligation into pNPTS-138-R6KT_P_*BAD*__*luxCDABE* as described above. HisL*_lux reporter strains (Supplementary data file S3) were generated by single homologous recombination as described previously^29^. Briefly, *E. coli* WM3064 cells were transformed with pNPTS-138-R6KT vectors^28^ carrying the *lux* operon preceded by either native or synthetic His-leader peptides (Supplementary data file S4). The vectors were transferred into the target *E. coli* BW25113 or Δ*efp* cells by conjugation. Transformants were selected from LB agar plates supplemented with kanamycin sulfate. PCR (Pf: HisL_chk_fw Pr: LuxC_chk_rev, Supplementary data file S5) and subsequent sequencing of the amplicon were used to verify incorporation of the correct hisL*.

tRNA deletion strains (Supplementary data file S3) were generated according to the “Quick and Easy *E. coli* Gene Deletion Kit by Red®/ET® Recombination” protocol (Gene Bridges). In short, primers containing 50 base-pair overhangs corresponding to the tRNA loci (Supplementary data file S5) were used to amplify linear FRT-side-flanked resistance cassettes from either FRT-PGK-gb2-neo-FRT or FRT-PGK-gb2-cat-FRT (Supplementary data file S4) using PCR. *E. coli* BW25113 cells transformed with pRED/ET were transferred from a thick overnight culture into a fresh culture in LB by 1:100 dilution, which was grown at 37 °C for about 2 h, until an optical density at 600 nm (OD_600_) of 0.3 was reached. Cells were then harvested and washed in 10% glycerol three times. The cells were subsequently transformed with the linear fragment by electroporation. Successful integration was confirmed by selective growth on LB plates containing either kanamycin sulfate or chloramphenicol and by PCR. Loss of the temperature sensitive pRED/ET plasmid was confirmed by selective growth on LB plates containing carbenicillin sodium salt or no antibiotic. To remove the chromosomally integrated resistance cassettes, the corresponding strains were transformed with the 707-FLPe plasmid (Supplementary data file S4) and transformants were subsequently inoculated in LB and grew at 30 °C for 2 h before shifting the temperature to 37 °C for overnight incubation. On the next day, cells were streaked out on LB plates and incubated overnight at 37 °C. Successful removal of the resistance cassettes and the temperature sensitive 707-FLPe plasmid was confirmed by selecting cells on plates with and without antibiotic and subsequent sequencing of the corresponding loci after colony PCR.

Plasmids for expression of *E. coli* tRNAs under control of their native promoters were generated by amplification of the corresponding genes and putative regulatory regions from *E. coli* BW25113 genomic DNA using specific primers (Supplementary data file S5) and subsequent cut/ligation into the pBBR1-MCS4-*lacZ* vector^29^ (Supplementary data file S4). Plasmids for expression of *E. coli* tRNAs under control of the *proL* promoter were generated using primers with a 70 BP overhang corresponding to P_*proL*_ (Supplementary data file S5) and subsequent cut/ligation into pBBR1-MCS4-*lacZ*.

Plasmids for quantification of ribosome slipping were generated by overlap extension PCR using primers with the sequence ATTAACCATGGGGNNN*TAG***G**ACTAAAAAAATTTCATTC (Supplementary data file S5) and pBAD_HisA-*luxCDABE*^27^ (Supplementary data file S4) as template. The first underlined sequence of the primer designates the initial open reading frame coding for a short peptide that stops at the TAG codon (italic). NNN designates the mutagenized region coding for the slipping sequence. The single base **G** (bold) allows the +1 frameshift into the *luxCDABE* open reading frame which is represented by the second underlined sequence.

### Growth conditions

*E. coli* cells were routinely grown in Miller modified Lysogeny Broth (LB)^45,46^ at 37 °C aerobically under agitation, if not indicated otherwise 1.5% (w/v) agar were used to solidify media when required. Antibiotics were added at the following concentrations: 100 µg/ml carbenicillin sodium salt, 50 µg/ml kanamycin sulfate, 20 µg/ml gentamycin sulfate. Plasmids carrying P_*BAD*_^47^ were induced with L-arabinose at a final concentration of 0.2% (w/v).

### Measurement of pausing strength in vivo

Pausing strength at PP-motifs was determined by measuring light output of the *lux* operon under the control of a synthetic His-leader peptide (HisL*) (Figs. 4c–6). Cells carrying the reporter were inoculated in 96-well plates (Sarstedt TC-Plate 96-Well, Standard d, F) with each well containing 200 µl of LB supplemented with kanamycin sulfate and incubated in an Eppendorf Thermomixer comfort at 37 °C and 550 rpm for at least 16 h. When expressing *E. coli* tRNAs from MCS4 plasmids, carbenicillin sodium salt was also added to the medium. On the next morning, Corning® 96-well flat clear bottom black polystyrene TC-treated microplates containing 200 µl of LB—supplemented with either kanamycin sulfate alone or in combination with carbenicillin sodium salt—were inoculated with 2 µl of overnight culture. The plates were directly transferred to a Tecan Spark® plate reader. Absorption at 600 nm (Number of flashes: 10; Settle time: 50 ms) and luminescence emission (Attenuation: none; Settle time: 50 ms; Integration time: 200 ms) were determined in between 10-min cycles of agitation (orbital, 180 rpm, amplitude: 3 mm) for around 16 h.

### β-Galactosidase activity assay

*E. coli* HisL* reporter strains (Supplementary data file S3) containing plasmids for expression of *E. coli* tRNA (Supplementary data file S4) were inoculated in 1.5 ml LB containing kanamycin sulfate and carbenicillin sodium salt and cultivated overnight in an Eppendorf Thermomixer comfort at 37 °C under microaerobic conditions and agitation at 650 rpm. On the next day, the optical density (OD_600_) was determined in 1 ml volumes containing 0.5 ml overnight culture and 0.5 ml of fresh LB medium. In total, 0.5 ml of overnight culture were transferred to a new 2 ml Eppendorf reaction tube. Cells were harvested by centrifugation and subsequently resuspended in 1 ml Buffer Z (0.06 M Na_2_HPO_4_, 0.04 M NaH_2_PO_4_, 0.01 M KCl, 0.001 M MgSO_4_). In total, 0.1 ml Chloroform and 0.05 ml 0.1 % SDS were added and the suspension was mixed by vortexing. Samples were preincubated at 30 °C for 5 min. The reaction was started by adding 0.2 ml of *ortho*-Nitrophenyl-β-galactoside solution (4 mg/ml in Buffer Z) and stopped by adding 0.5 ml 1 M Na_2_CO_3_ when yellow color formation was observed or after 5 min of incubation_._ The time between starting and stopping the reaction was noted in seconds. The samples were centrifuged at 20,000 × *g* for 10 min and 1 ml of the reaction solution was transferred to a cuvette. Absorbance at 420 nm was determined and Miller units (MU) were calculated as MU = 1000 × Abs_420_ × *t*^−1^ × *V*^−1^ × Abs_600_^−1,^^48^.

### Measurement of *cadBA* promoter activity in vivo

Activity of the *cadBA* promoter upon exchange of proline codons within the *cadC* gene (Fig. 7) was assessed using a luminescence reporter as described before^40^. *E. coli* MG1655 Δ*cadC* cells were cotransformed with the reporter plasmid pBBR1-MCS5-P_*cadBA*_-*lux* (Supplementary data file S4) and a pET16B vector for ectopic expression of either the wild-type *cadC* (pET16B-*cadC*) or a copy with silent mutations in the proline codon triplet CCU_P120_-CCC_P121_-CCU_P122_ leading to pET16B-*cadC_*3xCCG, pET16B-*cadC_*3xCCC, pET16B-*cadC_*3xCCU, and pET16B-*cadC_*3xCCA (Supplementary data file S4). As control the reporter plasmid was cotransformed with pET16B. Transformants were incubated in 200 µl of minimal medium developed by Epstein and Kim^49^ pH 7.6 supplemented with gentamycin sulfate, carbenicillin sodium salt, and 0.2% glucose (w/v) in 96-well plates in a Eppendorf Thermomixer comfort at 37 °C and agitation of 550 rpm overnight. On the next day, 2 µl of overnight culture were transferred to 200 µl of fresh medium supplemented with gentamycin sulfate and carbenicillin sodium salt in a Corning® 96-well flat clear bottom black polystyrene TC-treated microplate. Here, KE pH 5.8, 0.2% (w/v) glucose with varying concentrations of lysine was used. Bioluminescence emission (Attenuation: none; Settle time: 50 ms; Integration time: 200 ms) and growth (Wavelength: 600 nm; Number of flashes: 10; Settle time: 50 ms) were monitored in a Tecan Spark® in 10-min intervals during agitation (orbital, 180 rpm, amplitude: 3 mm) for around 16 h.

### Quantification of +1 translational frameshifting in vivo

*E. coli* BW25113 cells were transformed with plasmids containing pBAD-HisA-*luxCDABE* plasmids (Supplementary data file S4) in which the *luxC* gene was cloned out of frame as described above. In total, 200 µl LB containing the carbenicillin sodium salt were inoculated with 2 µl of an overnight culture of the desired transformants. To induce expression of the slipping vector arabinose was added to a final concentration of 0.2% (w/v). The measurement was performed in a Tecan Spark® reader in Corning® 96-well flat clear bottom black polystyrene TC-treated microplates. Bioluminescence emission (Attenuation: none; Settle time: 50 ms; Integration time: 200 ms) and growth (Wavelength: 600 nm; Number of flashes: 10; Settle time: 50 ms) were monitored in a Tecan Spark® in 10-min intervals during agitation (orbital, 180 rpm, amplitude: 3 mm) for around 16 h.

### Bioinformatic analyses

#### cDNA and protein sequences from *E. coli*

The cDNA and protein sequences of 4352 *E. coli* K-12 MG1655 genes were downloaded from the OMA database^50^. The cDNA and protein sequences of genes from the other 15 bacteria were downloaded from the Ensembl Bacteria database (Supplementary data file S1)^51^.

#### Identification of PP-motifs in protein sequences

PP-motifs in protein sequences were identified using the *fuzzpro* program from the EMBOSS package^52^. The PP-motifs were defined as in^11^, i.e., XX-*n*P-X where *n* ≥ 2 and X could be any non-proline amino acid.

#### Protein abundance and translation efficiency

We obtained the protein abundance and translation efficiency values for *E. coli* genes as described previously^11^: protein abundance data covering 2163 *E. coli* genes was from^53,54^; transcription levels of 2710 *E. coli* genes under standard growth conditions were downloaded from the ASAP database^55^. For each of the 1743 genes present in both datasets, we calculated the translation efficiency as the ratio between its protein abundance and transcription level.

#### Transmembrane segments of the *E. coli* proteins

Sequence positions of 5672 transmembrane segments within 912 α-helical transmembrane proteins were downloaded from the Uniprot database^56^. Data for the *E. coli* K-12 strain (taxonomy ID 83333) were used instead of *E. coli* K-12 MG1655 (taxonomy ID 511145), since the reviewed data of the latter are unavailable in the Uniprot database^56^.

#### Statistics and reproducibility

Sample size: sample size in biochemical experiments was chosen to be at least *n* = 4. This sample size was calculated from Lehr’s formula where the effect size was at least twice the standard deviation of experiments using wild-type cells. Biological replicates were defined as single colonies derived from culture plates. No data were excluded from the analysis. Replication: initial experiments using the His-Leader system (Fig. 4c) were conducted as technical replicates both in a Tecan Spark and a Tecan F500 reader showing qualitatively comparable results. Experiments on codon choice variation (Fig. 5b) were conducted both in 200 and 150 µl showing quantitatively comparable results.

### Reporting summary

Further information on research design is available in the Nature Research Reporting Summary linked to this article.

## Supplementary information

Supplementary Information

Description of Additional Supplementary Files

Supplementary Data 1

Supplementary Data 2

Supplementary Data 3

Supplementary Data 4

Supplementary Data 5

Supplementary Data 6

Reporting Summary

## Data Availability

The authors declare that the data supporting the findings of this study are available within the paper and its supplementary information. Source data underlying graphs presented in the main figures are available in Supplementary data file S6. No datasets were generated during this study.
