# Supplementary material for: Proline codon pair selection determines ribosome pausing strength and translation efficiency in bacteria
Source: Commun Biol. 2021 May 17;4:589. doi: 10.1038/s42003-021-02115-z (PMC8129111; doi:10.1038/s42003-021-02115-z)
Supplement: Supplementary file 1 — Supplementary Information [file 42003_2021_2115_MOESM1_ESM.pdf]

Proline codon pair selection determines ribosome pausing strength and translation efficiency in bacteria

Ralph Krafczyk[1]*, Fei Qi[2,3]*, Alina Sieber[1] , Judith Mehler[1] , Kirsten Jung[1], Dmitrij Frishman[3+] & Jürgen Lassak[1+]

[1] Department of Biology I, Microbiology, Ludwig-Maximilians-Universität München, Martinsried, Germany

[2] Institute of Genomics, School of Biomedical Sciences, Huaqiao University, Xiamen, China

[3] Department of Bioinformatics, Wissenschaftzentrum Weihenstephan, Technische Universität München, Freising, Germany

*These authors contributed equally to this work.

[+]To whom correspondence should be addressed. E-mail: d.frishman@wzw.tum.de or : juergen.lassak@lmu.de

Supplementary information

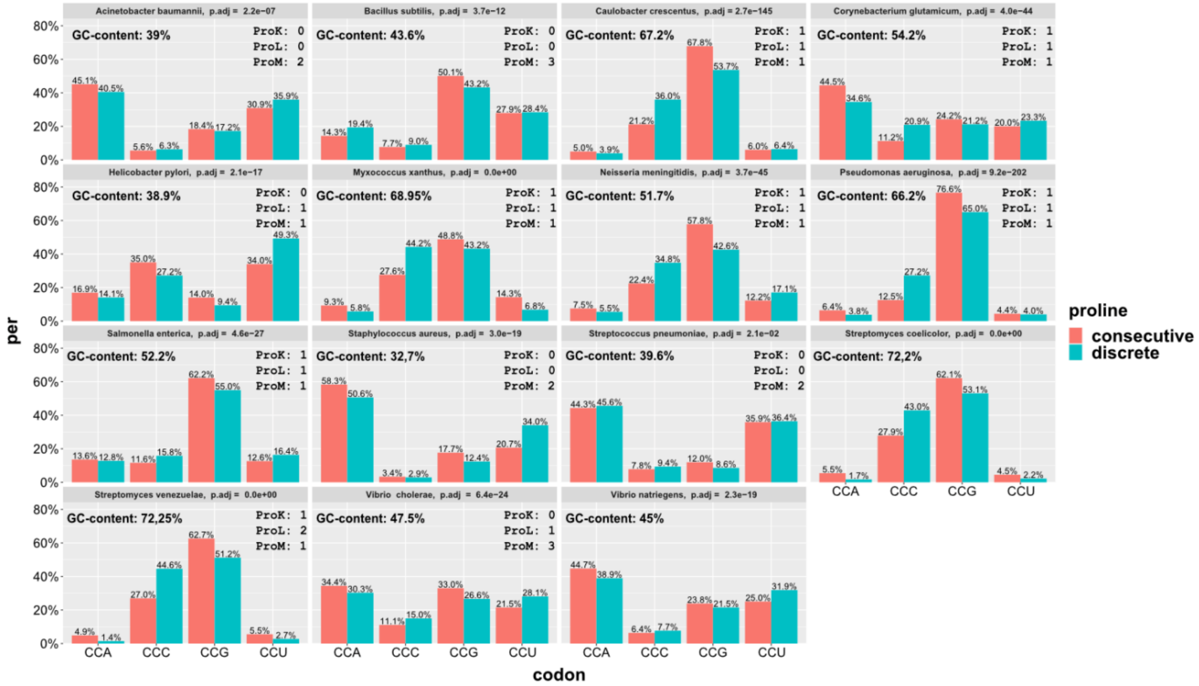

Fig. S1. Codon usage of single and consecutive prolines in the proteomes of 15 bacteria. The name of corresponding bacterium and the adjusted p-value of chi-squared test are shown in the title of each panel. The genomic GC-content and copy number of tRNA genes for individual bacteria are shown. tRNA copy numbers were derived from tRNA database (http://gtrnadb.ucsc.edu/)

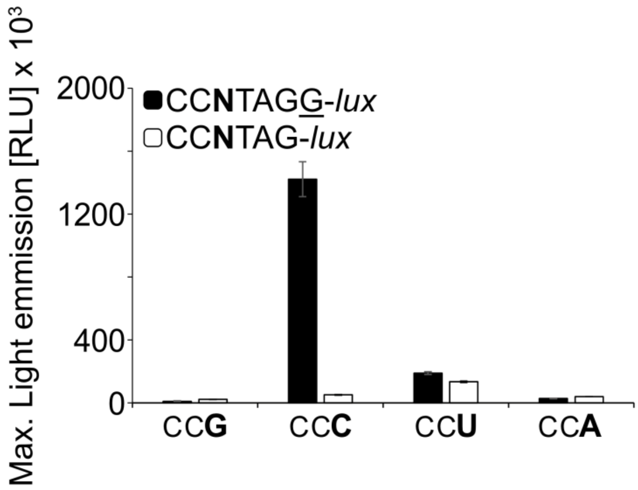

Fig. S2. Ribosome slipping and translational readthrough at proline codons. Maximal luminescence signals measured when the proline codons were coded in frame with the stop codon but in the -1 frame of the *lux*-operon (CC**N**TAGG-*lux*) are shown in black. Maximal luminescence signals measured when the proline codons were coded frame with the stop codon and the *lux*-operon (CC**N**TAG-*lux*) are shown in white.

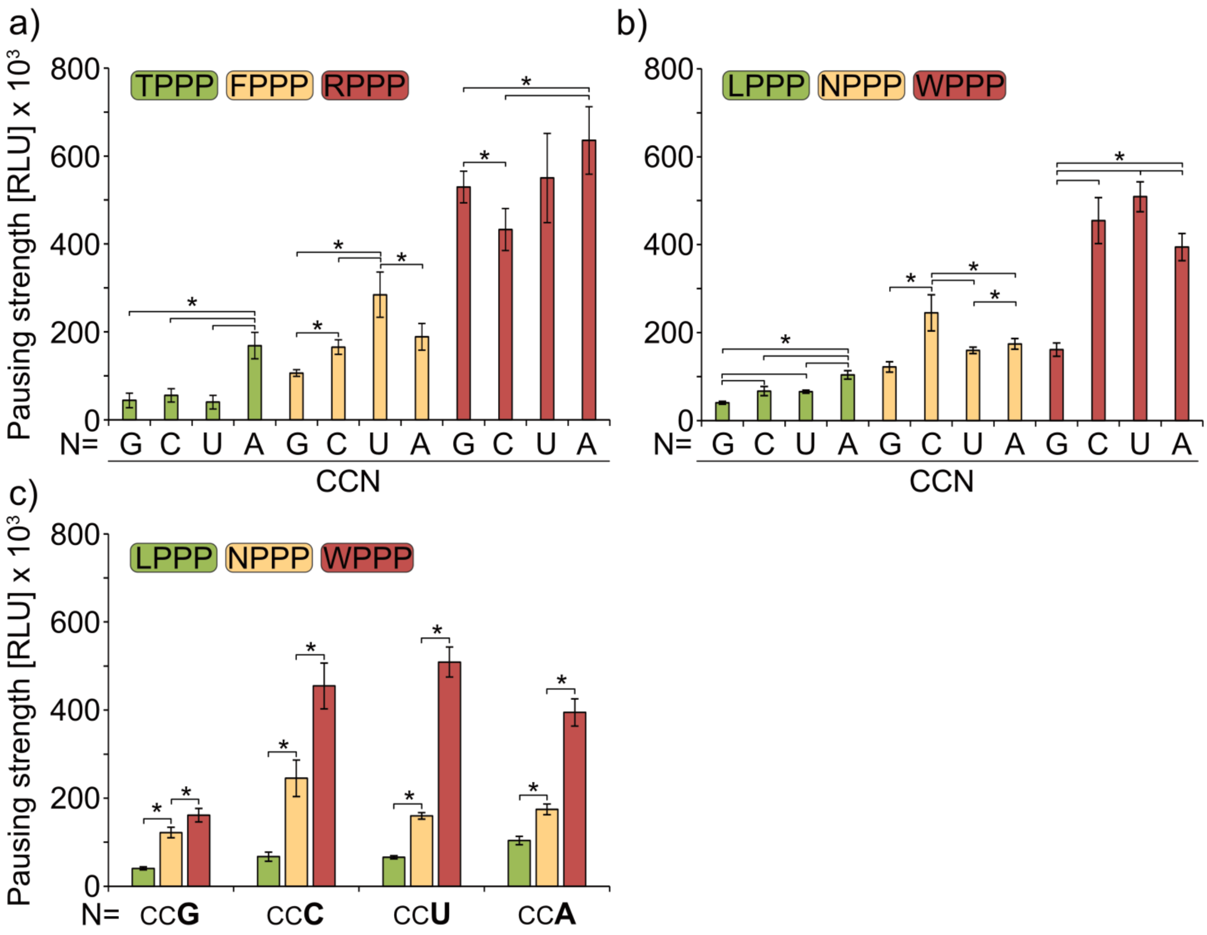

Fig. S3. Codon-dependent pausing strength at weak, intermediate and strong PP-motifs. HisL*_Lux carrying PP-motifs of varying pausing strength with different proline codon usage **A)** weak – TPPP: green; intermediate – FPPP: yellow; strong – RPPP: red; **B,C)** weak – LPPP: green; intermediate – NPPP: yellow; strong – WPPP: red) were chromosomally integrated in *E. coli* BW25113 and tested for maximal luminescence emission. n = 12, Error bars indicate 95% confidence intervals. Data for Figure 3A was duplicated from Figure 5B for better overview. Data for Figure 3C was duplicated from Figure 3B for better overview. Statistically significant differences according to unpaired two-sided t-tests (p-value < 0.05) are indicated by asterisks.

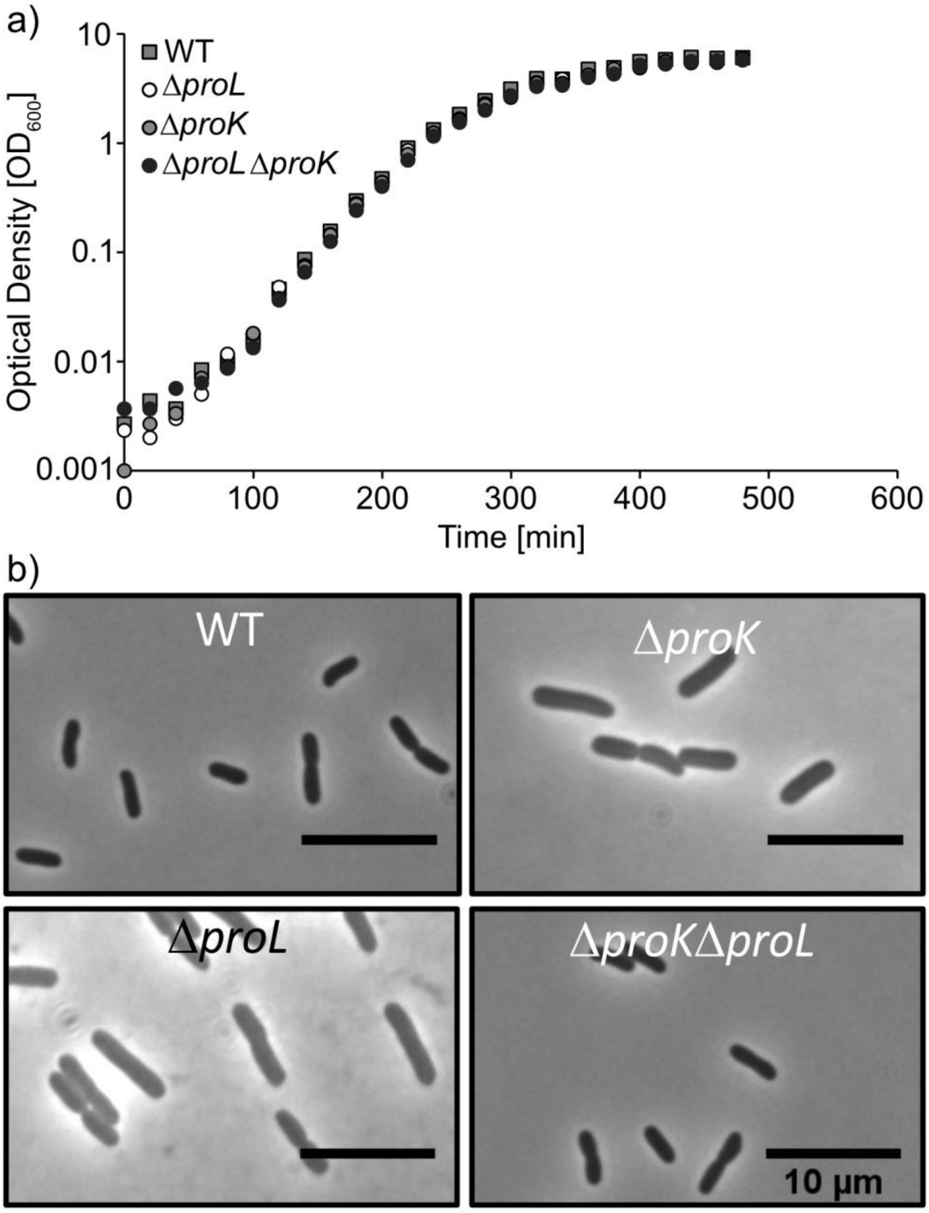

Fig. S4. Phenotypic characterization of *E. coli* BW25113 tRNA-deletion strains. A) Growth in LB medium at 37 °C under aerobic conditions. B) Phase contrast microscopy of exponentially growing cells.
