# Supplementary material for: Proline codon pair selection determines ribosome pausing strength and translation efficiency in bacteria
Source: Commun Biol. 2021 May 17;4:589. doi: 10.1038/s42003-021-02115-z (PMC8129111; doi:10.1038/s42003-021-02115-z)
Supplement: Supplementary file 2 — Description of Additional Supplementary Files [file 42003_2021_2115_MOESM2_ESM.pdf]

Description of Additional Supplementary Files

File name: Supplementary Data S1
Description: Oligonucleotides used during this study.

File name: Supplementary Data S2
Description: Plasmids used and generated during this study.

File name: Supplementary Data S3
Description: Strains used and generated during this study.

File name: Supplementary Data S4
Description: Bacterial genomes used for bioinformatic analysis.

File name: Supplementary Data S5
Description: Codon choice at di- and triprolyl motifs in E. coli

File name: Supplementary Data S6
Description: Source data underlying graphs presented in this study.
