# Supplementary material for: Proline codon pair selection determines ribosome pausing strength and translation efficiency in bacteria
Source: Commun Biol. 2021 May 17;4:589. doi: 10.1038/s42003-021-02115-z (PMC8129111; doi:10.1038/s42003-021-02115-z)
Supplement: Supplementary file 9 — Reporting Summary [file 42003_2021_2115_MOESM9_ESM.pdf]

nature research

Corresponding author(s): Jürgen Lassak
Dimitrij Frishman

Last updated by author(s): Mar 24, 2021

Reporting Summary

Nature Research wishes to improve the reproducibility of the work that we publish. This form provides structure for consistency and transparency in reporting. For further information on Nature Research policies, see our Editorial Policies and the Editorial Policy Checklist.

Statistics

For all statistical analyses, confirm that the following items are present in the figure legend, table legend, main text, or Methods section.

n/a	Confirmed	
☐	☒	The exact sample size (*n*) for each experimental group/condition, given as a discrete number and unit of measurement
☐	☒	A statement on whether measurements were taken from distinct samples or whether the same sample was measured repeatedly
☐	☒	The statistical test(s) used AND whether they are one- or two-sided<br>*Only common tests should be described solely by name; describe more complex techniques in the Methods section.*
☒	☐	A description of all covariates tested
☐	☒	A description of any assumptions or corrections, such as tests of normality and adjustment for multiple comparisons
☒	☐	A full description of the statistical parameters including central tendency (e.g. means) or other basic estimates (e.g. regression coefficient) AND variation (e.g. standard deviation) or associated estimates of uncertainty (e.g. confidence intervals)
☐	☒	For null hypothesis testing, the test statistic (e.g. *F*, *t*, *r*) with confidence intervals, effect sizes, degrees of freedom and *P* value noted<br>*Give P values as exact values whenever suitable.*
☒	☐	For Bayesian analysis, information on the choice of priors and Markov chain Monte Carlo settings
☒	☐	For hierarchical and complex designs, identification of the appropriate level for tests and full reporting of outcomes
☒	☐	Estimates of effect sizes (e.g. Cohen's *d*, Pearson's *r*), indicating how they were calculated

Our web collection on statistics for biologists contains articles on many of the points above.

Software and code

Policy information about availability of computer code

Data collection	No Software was used for data collection.
Data analysis	Identification of motifs in protein sequences: fuzzpro program from EMBOSS package version 6.6.0. Statistical analysis: R software version 4.0.2

For manuscripts utilizing custom algorithms or software that are central to the research but not yet described in published literature, software must be made available to editors and reviewers. We strongly encourage code deposition in a community repository (e.g. GitHub). See the Nature Research guidelines for submitting code & software for further information.

Data

Policy information about availability of data

All manuscripts must include a data availability statement. This statement should provide the following information, where applicable:

- Accession codes, unique identifiers, or web links for publicly available datasets
- A list of figures that have associated raw data
- A description of any restrictions on data availability

The authors declare that the data supporting the findings of this study are available within the paper and its supplementary information.
No datasets were generated during the current study.

Field-specific reporting

Please select the one below that is the best fit for your research. If you are not sure, read the appropriate sections before making your selection.

- [x] Life sciences
- [ ] Behavioural & social sciences
- [ ] Ecological, evolutionary & environmental sciences

For a reference copy of the document with all sections, see nature.com/documents/nr-reporting-summary-flat.pdf

Life sciences study design

All studies must disclose on these points even when the disclosure is negative.

Sample size	Sample size in biochemical experiments was chosen to be at least n = 4. This sample size was calculated from Lehr's formula where the effect size was at least twice the standard deviation of experiments using wildtype cells. In all experiments where n > 4, sample size was chosen based on technical parameters such as available wells in a 96 well plate.
Data exclusions	No data was excluded from the analysis.
Replication	Initial experiments using the His-Leader system (Figure 4C) were conducted as technical replicates both in a Tecan Spark and a Tecan F500 reader showing qualitatively comparable results. Experiments on codon choice variation (Figure 5B) were conducted both in 200 µl and 150 µl showing quantitatively comparable results.
Randomization	Experimental groups were formed by chosing samples with expectedly similar light output, so as to minimize the effect of residual light detection in neighbouring wells. Therefore, samples with the same preceeding amino acid (Figure 5B, Figure 6BCD) were measured in groups. In all other experiments, all samples were measured at the same time (Figure 7) or successively (Figure 6A) without grouping
Blinding	Blinding was not necessary for the biochemical part of this study, as all data was quantitatively determined using technical readouts of either light-emitting or colorimetric biochemical assays and no interpretation of any direct observation from the experimentator took place.

Reporting for specific materials, systems and methods

We require information from authors about some types of materials, experimental systems and methods used in many studies. Here, indicate whether each material, system or method listed is relevant to your study. If you are not sure if a list item applies to your research, read the appropriate section before selecting a response.

Materials & experimental systems

n/a	Involved in the study	
x		Antibodies
x		Eukaryotic cell lines
x		Palaeontology and archaeology
x		Animals and other organisms
x		Human research participants
x		Clinical data
x		Dual use research of concern

Methods

n/a	Involved in the study	
x		ChIP-seq
x		Flow cytometry
x		MRI-based neuroimaging
